# Effect of Exposure Conditions on Mortar Subjected to an External Sulfate Attack

**DOI:** 10.3390/ma17133198

**Published:** 2024-06-30

**Authors:** Othman Omikrine Metalssi, Marc Quiertant, Mike Jabbour, Véronique Baroghel-Bouny

**Affiliations:** 1 University Gustave Eiffel, Cerema, UMR MCD, F-77454 Marne-la-Vallée, France; mike.k.jabbour@gmail.com (M.J.);; 2 University Gustave Eiffel, EMGCU, F-77454 Marne-la-Vallée, France; mquiertant@estp.fr; 3 Institut de Recherche, ESTP, F-94230 Cachan, France

**Keywords:** external sulfate attack, exposure conditions, water–cement ratio, durability

## Abstract

This study aims to investigate the influence of exposure conditions on the behavior of mortar subjected to an external sulfate attack (ESA). Three different exposure conditions (full immersion, semi-immersion, and drying/wetting cycles) were tested on mortar prisms made with Portland cement and two w/c ratios (0.45 and 0.6). To monitor degradation, it was necessary to evaluate variations in length (expansion), mass changes, compressive and tensile strengths, changes in the total porosity measured using water accessible porosity tests, and changes in the macroscopic behavior of the samples. Mercury intrusion porosimetry (MIP) was used to determine the size distribution of the pores. It was demonstrated that mixing mortar with the lower w/c ratio of 0.45 results in improved performance against an ESA. This study also demonstrates that the type of exposure to an ESA has no significant effect on the kinetics of sulfate penetration during the exposure period. However, the sample’s surface becomes more cracked when subjected to repeated drying and wetting cycles. For all the considered exposure conditions, expansion occurred in three stages. In stage 1, the reaction product (ettringite) precipitated in large voids, without causing significant expansion (the expansion remained low and stable). During the second stage, the reaction products generated growing internal stress. The final stage of expansion resulted in microcracks, strength losses, and the formation of macropores, which ultimately lead to material failure. The MIP results indicate that major changes in the porosity and pore volume distribution occur at the surface layer in regard to the gel and capillary pore ranges.

## 1. Introduction

Various forms of deterioration can occur in reinforced concrete (RC) structures, including an external sulfate attack (ESA), which can adversely impact the durability and integrity of the structure [1,2]. Such an attack occurs when sulfates from external sources, such as soil or groundwater, react with the components of concrete, resulting in the formation of expansive compounds. As a result of sulfate ingress into the concrete matrix, ettringite crystals are formed [3,4]. Consequently, the concrete expands, cracks, spalls, and loses its structural integrity. The sulfate concentration, exposure conditions, and concrete quality can all influence the severity of these signs of deterioration [5]. In order to develop effective mitigation strategies and ensure the long-term performance of concrete elements exposed to sulfate-rich environments, it is crucial to understand the mechanisms and manifestations of this degradation process [6,7,8]. The service life of RC structures can be extended by taking appropriate measures and adopting monitoring techniques to minimize the detrimental effects of ESAs.

The effect of ESA-related degradation can be significantly influenced by the exposure conditions and the water-to-cement (w/c) ratio. The exposure conditions, such as the type and concentration of sulfates, as well as the humidity and temperature, play an important role in the progression of a sulfate attack [5]. Generally, higher temperatures increase the rate of sulfate ingress and expansion within concrete pores, thus accelerating the degradation process. Concrete can deteriorate at high humidity levels due to sulfate penetration and chemical reactions. There is a link between more severe damage and higher sulfate concentrations and longer exposure times [5].

An increase in the w/c ratio can increase the permeability of concrete mixtures and make them more susceptible to sulfate infiltration [9,10]. The presence of excessive water in concrete allows sulfates to penetrate the matrix more easily, resulting in chemical reactions that accelerate the expansion and cracking of the concrete. Contrary to this, concrete with a lower w/c ratio is denser and more resistant to an ESA [9]. However, due to the rapid filling of the porosity by the expanded product formed, the damage to concrete in this case may be sudden and more serious [8].

The impact of exposure conditions, such as total immersion, partial immersion, and wet–dry cycles, on the development of an ESA in concrete, is an important research topic for ensuring the durability of concrete structures [8,11,12,13]. It is also a subject of ongoing debate among scientists. Different exposure modes can have a significant effect on the progression and severity of an ESA, which necessitates a thorough analysis to better understand the underlying mechanisms. Despite some studies suggesting a clear correlation between these exposure conditions and the severity of an ESA, others present conflicting findings, leading to disagreement within the scientific community [11,12,13,14,15,16,17,18,19]. These studies have demonstrated that prolonged total immersion of concrete in sulfate-rich environments can lead to rapid degradation of the material, due to deep sulfate penetration and chemical reactions. A concrete structure immersed in a sulfate-containing solution is particularly prone to deterioration over time. The results confirm that total immersion causes more significant damage than partial immersion. However, Hartell et al. [20] challenge this assertion, stating that partial immersion may also cause substantial deterioration under certain conditions.

Similarly, research by Zhang J. et al. [21] has highlighted that partial immersion, where only a portion of the concrete is exposed to sulfate-rich water, can also result in significant damage, although generally less severe than observed with total immersion. The uneven distribution of sulfate ingress in partially immersed structures may result in localized damage and surface deterioration. Regarding wet–dry cycles, characterized by alternating periods of wetting and drying, investigations carried out by Wang et al. [22] and Liu et al. [23] have demonstrated that constant moisture variations can promote sulfate migration through concrete pores, accelerating the ESA process and causing structural damage more rapidly than under static conditions. The repeated absorption and desorption of moisture can promote sulfate penetration into the concrete and enhance the chemical reactions. It is also important to note that conflicting views exist regarding this specific exposure condition, which can occur in tidal zones. According to Chabrelie [24], wet–dry cycles involve an oscillation of the relative humidity in the material. This leads to a delay in the chemical fixation of sulfate ions, which highlights the sensitivity of hydrates to drying. Moreover, wet–dry cycles can induce a protective mineral layer that may mitigate an ESA.

These discrepancies illustrate the complexity of ESA mechanisms and emphasize the need for further investigation to determine the precise effect of exposure conditions on concrete durability. This article aims to provide appropriate answers in response to these debates by conducting comprehensive experiments and analyses on mortar cast with Portland cement to gain a better understanding of how different exposure conditions, including complete immersion, partial immersion, wet–dry cycles, and water-to-cement ratios, influence the development of an ESA. Investigations are conducted at the microstructural, macrostructural, and mechanical levels.

## 2. Materials and Methods

### 2.1. Formulations and Exposure Conditions

Portland cement (CEM I 52.5 N CE CP2 NF) produced by EQIOM (Rochefort, France) was used to cast the mortar prisms, with dimensions of 4 × 4 × 16 cm^3^, and the chemical composition is given in Table 1. Inductively coupled plasma–atomic emission spectroscopy (ICP-AES) from Analytik Jena GmbH (Jenna, Germany) and thermogravimetric analyses (TGA) form a NETZSCH STA449 F1 instrument (Selb, Germany) were combined to obtain this composition. In Table 2, the contents of the clinker are summarized according to Bogue’s method [25]. It appears that this clinker contains significant amounts of aluminate in the form of C_3_A and C_4_AF. The mortar samples were prepared using ordinary sand from the Palvadeau quarry in France, which is 98% siliceous by weight, with natural sand ranging from 0 to 4 mm, and which has a relative density of 2640 kg/m^3^. The mixture proportions used for manufacturing were the ones used for standard mortars, namely 1350 g of sand and 450 g of cement, with two w/c ratios (0.45 and 0.60). These water-to-cement ratios were carefully considered to ensure proper hydration and strength development.

**Figure 1:**
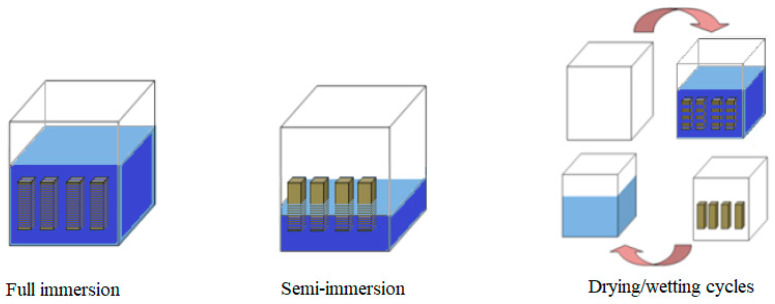
The three exposure conditions considered for ESA tests.

Immediately following demolding, the mortar samples were placed in large tanks filled with tap water to undergo a moist cure for 28 days. As mentioned above, the effect of the water content on the resistance against an ESA has been taken into consideration in this part of the research, by selecting a high w/c ratio of 0.6 and a moderate w/c ratio of 0.45 as examples. A study was conducted to determine how the exposure environment affected the interaction between the sulfates and cement-based materials under three different conditions (Figure 1). A comparison between the three testing methods could also be useful in identifying the most suitable method that results in significant deterioration within a short period. There were three exposure settings: full immersion, semi-immersion, and drying/wetting cycles (3 days of wetting, and 4 days of drying).

During the experiment, sodium sulfate solution Na_2_SO_4_ was used at a concentration of 15 g/L, without pH control. For the first month, 1/3 of the solution was renewed every week, every two weeks for the second and third months, and every week for the remainder of the study. For the full immersion and drying/wetting cycles, the volume of the sulfate solution was greater than 8.5 times the volume of the prisms, whereas for the semi-immersion cycle, this ratio was approximately 3.5 times the volume of the prisms.

Finally, a simple nomenclature was used to designate the samples according to the type of material studied (here, only “M I” for mortar made with cement CEM I), namely the w/c ratio (0.45 or 0.6) and the type of exposure to an ESA (“Imm” for total immersion, ”Semi-Imm” for semi-immersion, or “Cycles” for drying/wetting cycles). For example, “M I-0.6 Imm” means: a sample of mortar made of ordinary cement CEM I, with a w/c ratio of 0.6, and completely immersed in the sulfate solution during the attack.

### 2.2. Experimental Techniques for Investigation

Visualizations of the samples were conducted to demonstrate the progression of deformations and cracks caused by an ESA. Visual examinations can provide valuable insight into the structural response of the material to an ESA over time and can identify and characterize any changes that have occurred in the morphology and integrity of the sample over time.

**Figure 2:**
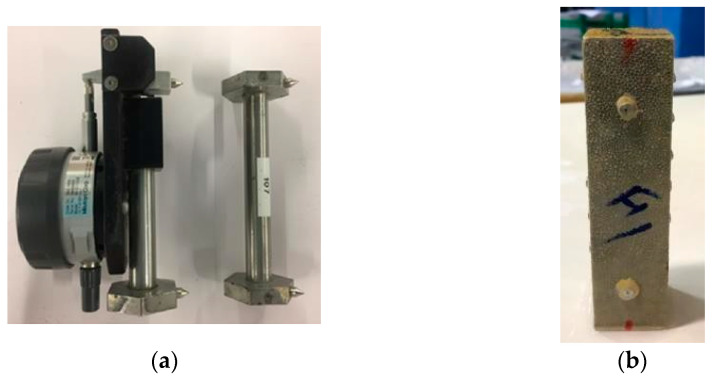
The extensometer and calibration section used to measure the variation in length (**a**); the mortar prism with a pair of pins fixed on one of the sides (**b**).

The expansion response of hardened mortar samples to an ESA is primarily assessed by recording the continuous change in the length of the samples. An extensometer and a steel reference length bar are used to record the variation in length between two pairs of stainless-steel pins (Figure 2a). Pins are adhered to the samples after demolding, using strong chemical glue that is resistant to a chemical attack in liquids, such as sulfate solutions. The pins are equipped with a tiny hole on the top, which corresponds to the pointers on the extensometer and the reference length bar. A generating line was formed by keeping each pair of pins aligned in the middle axis of the prism’s face, at a length of 100 mm (Figure 2b). As with the expansion measurements, the mass variation was measured using the same mortar prisms.

During the exposure to an ESA, the compressive and tensile strength of the mortar samples were determined using STDME172-01 Apparatus from SMART TESTING AND DRILLING EQUIPMENTS (Eaubonne, France). The tests were conducted at different time intervals: 28 days after the moisture cure for reference, and 3 months, 6 months, 9 months, and 12 months after ESA exposure. In the first step, the prisms were subjected to a three-point load test. Then, compressive strength tests were conducted, using the two parts obtained from the flexural strength test. Each measurement was performed on three samples for bending and 6 cubes for compression. The standard deviation of the average measured value was calculated for each type of test. For the semi-immersion samples, “semi-drying” is called for in regard to the part of the sample that remains exposed to the air.

The porosity and microstructural changes in the mortar samples were assessed using water accessible porosity tests (WAPTs) and mercury intrusion porosimetry (MIP) analyses, using the Micrometrics Autopore IV 9520 Porosimeter instrument (Villiers sur Marne, France), at different stages. WAPTs provide valuable information regarding the volume of interconnected pores within mortar that can be infiltrated by water, providing insights into the material’s susceptibility to moisture-related damage. The MIP method, on the other hand, is a powerful method for measuring pore sizes in mortar, by using the mercury intrusion pressure to examine the pore network at the microstructural level.

Finally, a total of six “Experimental Units”, three exposure conditions (total immersion, semi-immersion, cycles) and two water-to-cement ratios (0.45 and 0.60), were considered in this study. As for the “Evaluated Units”, the properties measured include porosity and pore size distribution, mechanical strength, visual observations, mass, and expansion. Therefore, for each experimental unit (e.g., the specific water-to-cement ratio and exposure condition combination), these properties were evaluated and recorded. For each “Evaluated Unit” and time, three samples were used in order to find the mean value, with the standard deviation. A total of 108 samples (4 × 4 × 16 cm^3^) were manufactured for all the tests.

## 3. Results

### 3.1. Visual Inspections

**Figure 3:**
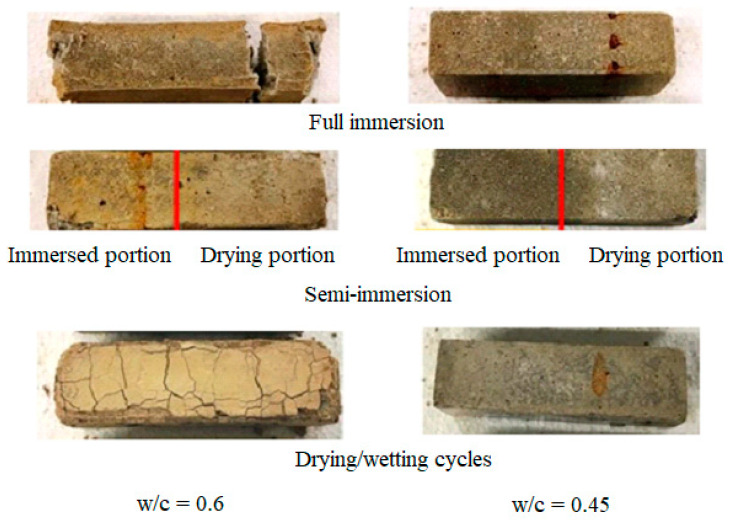
Visual appearance of the samples after 12 months of exposure to sulfate solution. The red line in the semi-immersion exposure separates the immersed and the drying portions.

Figure 3 shows the external appearance of the mortar samples after 12 months of exposure to an ESA. Three samples were inspected for each w/c ratio and each exposure condition. The mortar prisms with w/c = 0.45, placed in full immersion, partial immersion, and drying/wetting cycles, in 15 g/L of Na_2_SO_4_ solution, did not exhibit significant degradation after 12 months. There was some sort of spalling around the edges and minor surface scaling observed for the three exposure conditions. Nevertheless, the mortar samples with w/c = 0.6 were found to be so damaged that they broke when removed from the bath. All kinds of cohesion were lost, and they showed longitudinal and transverse cracks, as well as significant swelling at the edges. Therefore, internal tensile stresses were suggested to be responsible for the total damage and loss of material observed during full immersion.

The mortar prisms with a higher w/c ratio showed clear signs of degradation after 12 months of semi-immersion. Longitudinal and transverse cracks appeared at the edges, while the surface of the samples disintegrated. The degree of deterioration varied between the upper portions exposed to the surrounding atmospheric conditions (temperature and relative humidity) and the lower portions submerged in the Na_2_SO_4_ solution. Crystallization of salt, in the form of white sodium sulfate crystals, was clearly evident during the drying process. It is believed that crystals form through capillary absorption, which causes the liquid solution containing sulfate ions to move upward from the immersed to the drying portion of the solution. Salt crystals develop directly above the sodium sulfate solution as a result of temperature and relative humidity changes [26]. Meanwhile, the immersed parts were severely damaged and became loose as a result of circumferential cracks appearing on the top surfaces of the samples, along with pitting damage, scaling at the edges, and the loss of material and particles.

The inspection of the mortar prisms during the drying/wetting cycles with a higher w/c ratio revealed serious deterioration, with the appearance of longitudinal and transverse cracks at the edges and corners. Interestingly, the monitored samples suffered from cracks expanding in all directions at the level of the surface exposed to air during casting.

Increasing the w/c ratio from 0.45 to 0.6 appears to accelerate and increase the severity of the damage mechanisms. As shown by the different degradation mechanisms, the damage process during an ESA is dependent on the porosity of the cementitious material, which is related to the amount of water content. It is believed that the interaction between the penetrating sulfate ions and the microstructure of the material may affect the mechanical response.

### 3.2. Expansion and Mass Variation

**Figure 4:**
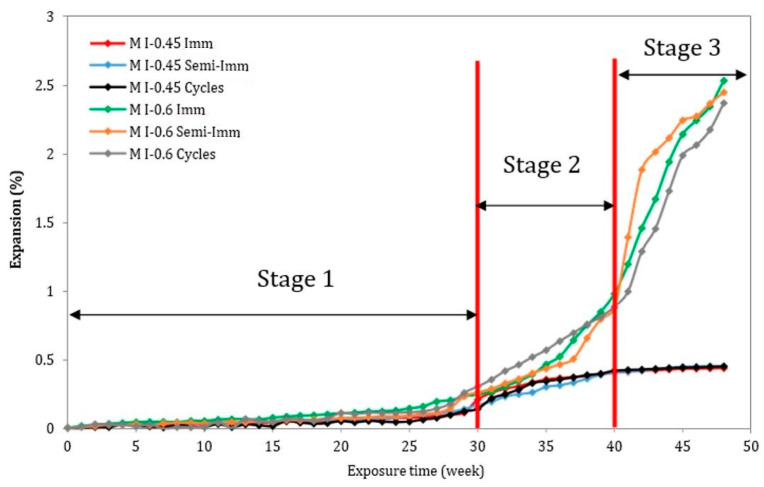
Evolution of expansion of mortar samples following the three-stage process.

Figure 4 illustrates the expansion results of the mortar prisms with the two considered w/c ratios (M I-0.45 and M I-0.6). For the M I-0.6 samples, an important increase in expansion was observed, regardless of the exposure conditions. As a result of the full immersion, M I-0.6 Imm showed the highest average final expansion (2.2%), which was reached at week 48. The M I-0.6 Semi-Imm and M I-0.6 Cycles samples showed final expansions of 2.1% and 2%, respectively. Conversely, all the mortar mixes cast with w/c = 0.45 (M I-0.45 Imm, M I-0.45 Semi-Imm, and M I-0.45 Cycles) revealed a small average expansion of 0.4%. These results indicate that a higher w/c ratio is associated with greater expansion. The higher the w/c ratio, the more porous the structure becomes, allowing sulfate ions to enter and to react freely. Interestingly, the kinetics and amplitude of expansion during an ESA are very close whatever the exposure conditions, but with the w/c ratio having a pronounced effect. The expansion rates of the M I-0.45 prisms followed the same trend under full immersion, semi-immersion, and drying/wetting conditions.

As illustrated in Figure 4, the evolution of expansion seems to follow a three-stage behavioral process: stage 1 with low and stable expansion (from week 0 to week 30), which corresponds with the preliminary phases of the attack, when sulfate ions start to diffuse into the cement matrix to react with cement hydrates and aluminate sources to form expansive products (ettringite) without generating expansion stresses; stage 2 with significant expansion without causing severe macroscopic damage (from week 30 to week 40), during this period the precipitation of the new products inside the empty pores start to create stresses that lead to elevated rates of expansion and the formation of microcracks, but without expressing any visual signs of macroscopic deterioration; and stage 3 with destructive expansion leading to failure (after week 40). During this last period, cracks begin to develop and become larger and wider, which increases the amount of sulfate ions that enter the material through the microstructure of the crack. As a result, the attack becomes more aggressive as ettringite forms inside the new cracks, causing the expansion to suddenly increase before the cracks’ collapse completely. This process is apparent for the three types of exposure.

**Figure 5:**
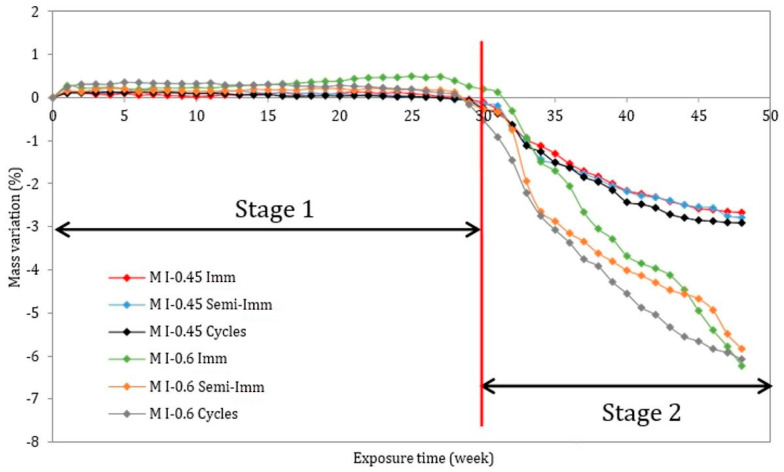
Mass variation of the mortar samples.

As for the variation in mass, the mortar samples exposed to three different accelerated attacks (full immersion, semi-immersion, and drying/wetting cycles) began losing mass after almost 30 weeks of contact with the attacking solution (Figure 5). A small increase in the initial mass of the samples was followed by an important mass loss, particularly near the end of the experiment. The mass loss is primarily due to material loss, as particles began to break away from the prisms. The mass loss was more significant for the M I-0.6 samples which expanded more and experienced severe macroscopic damage. Accordingly, the mass variations can be divided into two stages. Week 30 marks the beginning of the switch from one phase to the next. During the first stage (stage 1), the mass increases slightly due to the precipitation of ettringite. This mass uptake is related to the molar volume of these newly formed expansive products, which is three times greater than the molar volume of AFm and portlandite. The first signs of mass loss, in stage 2, are believed to be caused by the dissolution of portlandite and the decalcification of C-S-H, which produce more calcium ions in the cement matrix. When more calcium is present, it becomes possible to produce gypsum and later ettringite as the Ca^2+^ interacts with the sulfate ions [27]. Interestingly, stage 2 of the mass variation was characterized by severe mass loss around week 30, which corresponds to the exact time when the expansion began to appear.

Finally, in stage 2 of the external sulfate attack, when mortar undergoes drying and wetting cycles it can experience high weight loss and swelling. This phenomenon is often attributed to the sample going from a dry to a wet state, which corresponds to the generation of a large amount of AFt in the mortar. In these conditions, sulfate ions can easily penetrate the material and react with the tricalcium aluminate (C_3_A) in the cementitious material to form a high amount of AFt. This leads to an expansive reaction that can lead to swelling. Indeed, repeated wetting and drying cycles can alter the pore structure of the mortar, leading to changes in its mechanical properties and durability. The increased porosity resulting from ettringite formation and dissolution can enhance water penetration and ion transportation within the material, further exacerbating degradation mechanisms. Moreover, during wetting and drying cycles, the ettringite formed in the mortar can undergo hydration and dehydration processes. When the mortar is wetted, ettringite can dissolve and reprecipitate upon drying. These cycles of dissolution and reprecipitation can contribute to the high weight loss and swelling observed in the mortar.

### 3.3. Compressive and Tensile Strength

**Figure 6:**
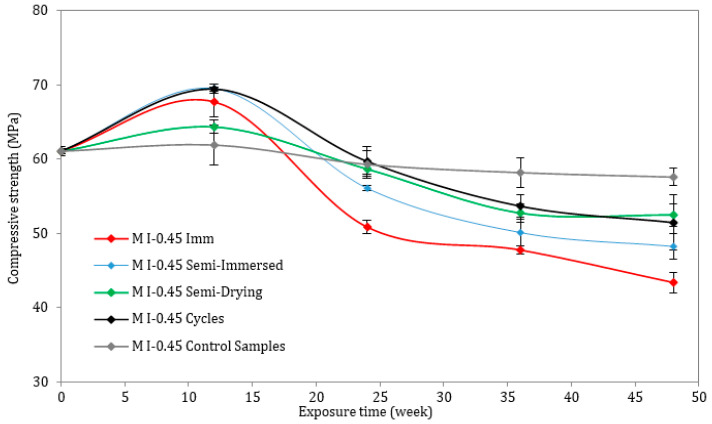
Compressive strength of mortar samples exposed to ESA or stored in water (w/c = 0.45).

**Figure 7:**
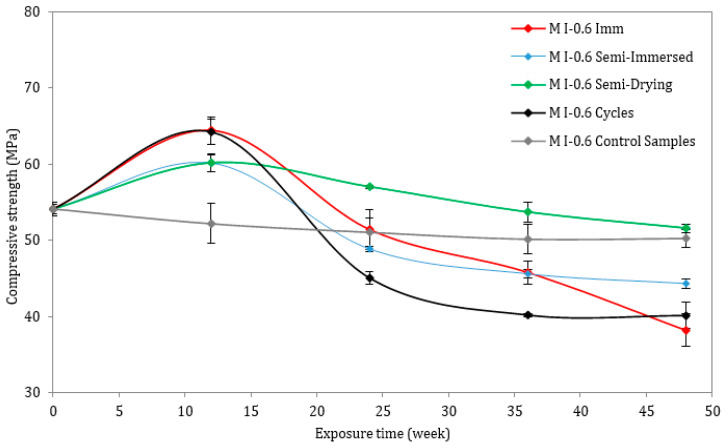
Compressive strength of mortar samples exposed to ESA or stored in water (w/c = 0.60).

This section presents the evolution of the compressive strength of all the mortar mixes at ages 0, 12, 24, and 48 weeks. The compressive strength of the reference (control) samples, immersed in tap water for 12 months, was also determined at the same ages. For both w/c ratios of 0.45 and 0.6, it is evident that the evolution of the compressive strength of mortar followed a two-stage behavior process. Considering the lower w/c ratio (Figure 6), the compressive strength increased from 61 MPa to a peak value after 3 months (12 weeks), and then it gradually decreased. In comparison with other types of accelerated attacks, samples under full immersion (M I-0.45 Imm) suffered the greatest strength loss, declining from 67 MPa after 3 months to 43 MPa after 12 months. In the case of the semi-immersed samples, both the dried and wet parts were measured for compressive strength (the wet part is labelled “Semi-immersed”, while the dried part is labelled “Semi-dried” in the figures). There was a greater loss of compressive strength in the submerged portion (lower value is 49 MPa), marked in blue, than in the drying portion (lower value is 54 MPa), marked in green, which is most likely due to the greater exposure to the chemical attack by the submerged portion. As it can be seen in Figure 7, regardless of the type of exposure, the compressive strength increased at different rates for the materials with a higher w/c ratio after 3 months. In all the samples, except the control samples, a significant decrease occurred after 6 months. At 12 months, the strength of the M I-0.6 Imm and M I-0.6 Cycles samples dropped from 64 MPa at 3 months to 38 MPa (M I-0.6 Imm) and 40 MPa (CEM I-0.6 Cycles), respectively.

The compressive strength results demonstrate that strength loss is significant with increasing w/c ratios. As a result of the precipitation of the first amount of ettringite produced inside the pores, the compressive strength increases during the first three months of exposure to the Na_2_SO_4_ solution. As a result of these products, the microstructure of the cement matrix is compacted and improved, resulting in a sudden increase in strength [28]. Upon exposure to an ESA for six months, the products began to affect the pore walls, exceeding the pore limit and creating expansive stresses exceeding the tensile strength of the mortar samples. Consequently, microcracks form and grow wider and larger, resulting in a significant decrease in the overall compressive strength [29]. Finally, the loss in compressive strength for both w/c ratios was greater for fully immersed samples than for semi-immersed samples and for samples subjected to drying/wetting cycles.

**Figure 8:**
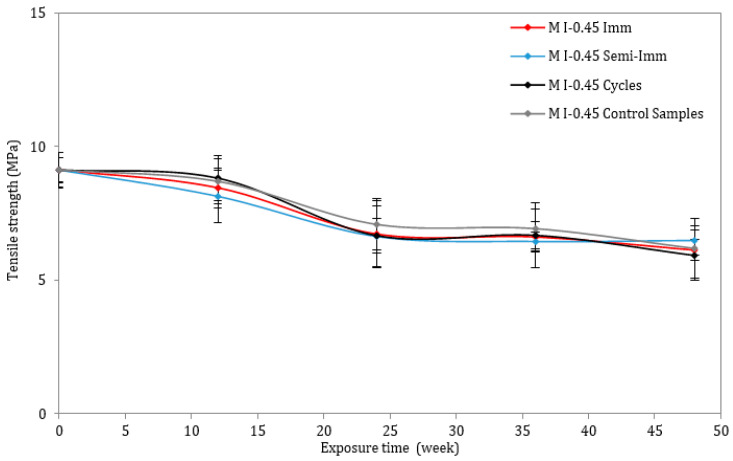
Tensile strength of mortar samples exposed to ESA or stored in water (w/c = 0.45).

**Figure 9:**
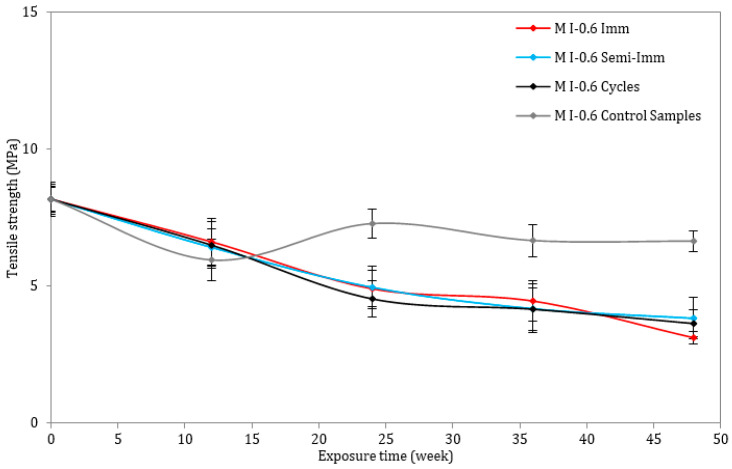
Tensile strength of mortar samples exposed to ESA or stored in water (w/c = 0.60).

On the other hand, the tensile strength of all the mortar mixes was determined through the use of three-point bending tests. Figure 8 and Figure 9 illustrate the loss rates concerning the tensile strength of the samples. The tensile strength decreased for both mixes, but the drop was more pronounced for the M I-0.6 samples following 12 months of immersion, semi-immersion, and drying/wetting cycles. The tensile strength decrease can be explained by some mechanisms, such as the increased porosity, microcracking, internal stresses, volume changes, and alterations in the pore structure. Indeed, the formation of ettringite is associated with an expansive reaction that can lead to the creation of additional pores and voids within the mortar matrix. This increase in porosity can weaken the interfacial bond between the cementitious materials, reducing the overall tensile strength of the material. The expansion associated with the crystallization of ettringite crystals can induce mechanical stresses in the surrounding matrix, leading to the initiation and propagation of cracks. These microcracks act as stress concentration points, reducing the load-carrying capacity of the material and compromising its tensile strength.

### 3.4. Porosity

**Figure 10:**
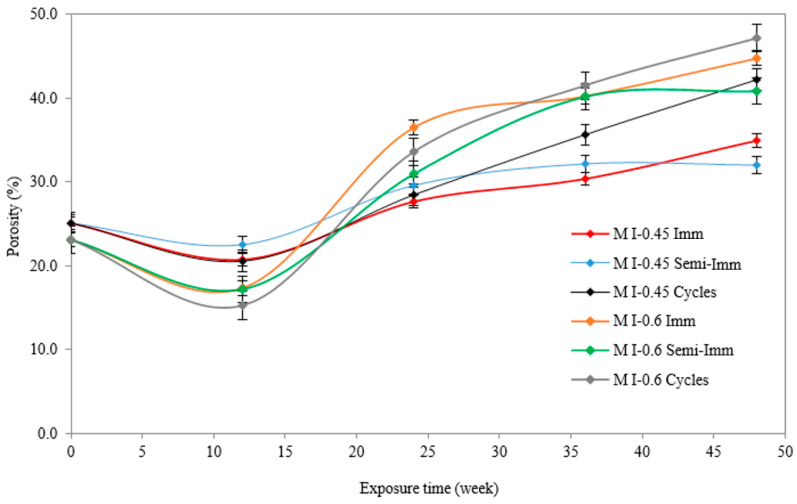
Total porosity of mortar samples due to ESA under three exposure conditions.

Figure 10 shows the distribution of porosity accessible to water for both w/c ratios and all exposure conditions. It is worth noting that this method provides the total number of open pores (micro, meso, and macropores) and reflects the permeability state of the material. In the presence of high porosity, the cement matrix is subjected to excessive penetration by sulfate ions through the open connected porosity. As a result, the precipitation of the reaction products formed during the ESA becomes more significant and faster inside the material, resulting in total material damage. Moreover, hydrostatic weighing determines the porosity based on the pore continuity and microcracks within the material [30].

The porosity percentage changed following a two-stage process. In the M I-0.6 samples, the porosity decreased from 23.12% before any contact with the Na_2_SO_4_ solution to 17.37% after 3 months (14 weeks) of full immersion, 17.24% after 3 months of semi-immersion, and 15.29% after 3 months of drying/wetting cycles. During the period of three months, the porosity of the M I-0.6 samples decreased by 39%, on average. On average, after 12 weeks of exposure to an ESA under the three conditions, the porosity percentage of the M I-0.6 samples decreased by 28%. Ettringite, as well as sulfate crystallization, results in the filling of pores [28]. It has been suggested that the sudden increase in porosity observed afterwards may be due to the release of expansive stresses by sulfate attack products, together with the excessive stresses exerted during the crystallization of sulfate, which affect the pore walls and create more empty spaces. The porosity percentages of the M I-0.6 mortar samples increased by 47.7% after 48 weeks of ESA. The presence of such a high porosity percentage makes the microstructure looser and leads to microcrack formation. The precipitation of ettringite within the material reduces pores and limits their connectivity. A significant increase in porosity occurred after 12 months, as high porosity percentages were found for the samples undergoing full immersion M I-0.6 Imm (44.73%), semi-immersion M I-0.6 Semi-Imm (40.82%), and drying/wetting cycles M I-0.6 Cycles (47.14%). Microcracks induced by excessive expansion stresses exerted by the reaction products allow more sulfate ions to diffuse into the cement matrix, causing more damage and increasing the total number of pores (cracks). The results for the M I-0.45 samples showed a similar trend to the M I-0.6 samples, but at lower percentage rates and amplitudes. After three months of exposure, the increase is believed to be the result of the decalcification of C-S-H in the cement matrix, which causes more Ca^2+^ to interact with sulfate ions and produce gypsum and then ettringite [27].

### 3.5. Pore Size Distribution

**Figure 11:**
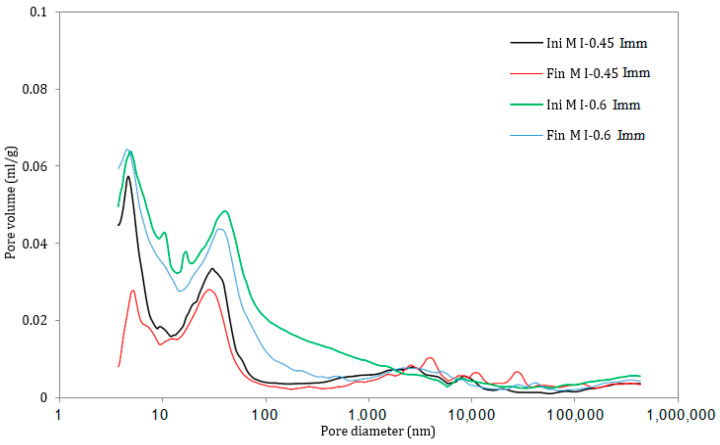
Pore size distribution in the mortar samples under full immersion, before (Ini) and after (Fin) ESA.

**Figure 12:**
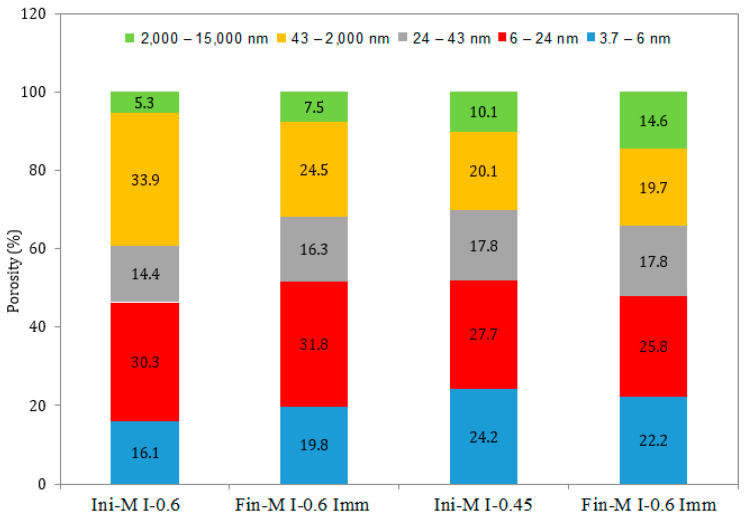
Variation in the pore volume of the samples during full immersion, before (Ini) and after (Fin) ESA.

**Figure 13:**
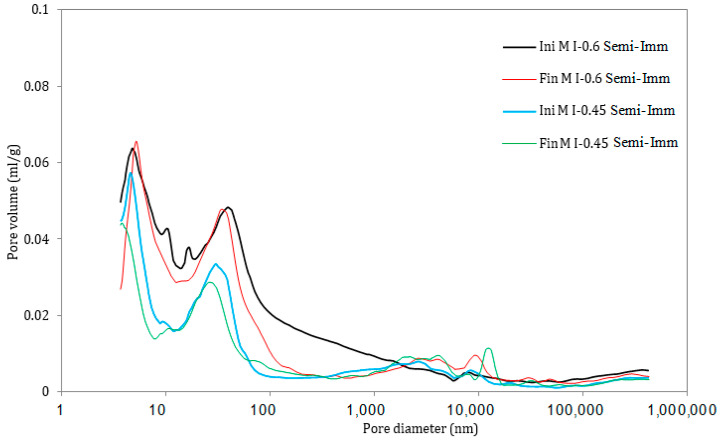
Pore size distribution in the samples under semi-immersion, before (Ini) and after (Fin) ESA.

**Figure 14:**
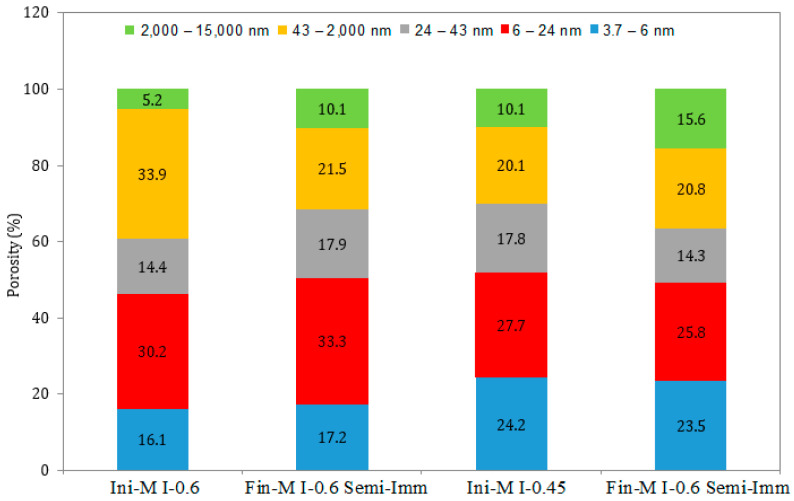
Variation in the pore volume of the samples during semi-immersion, before (Ini) and after (Fin) ESA.

**Figure 15:**
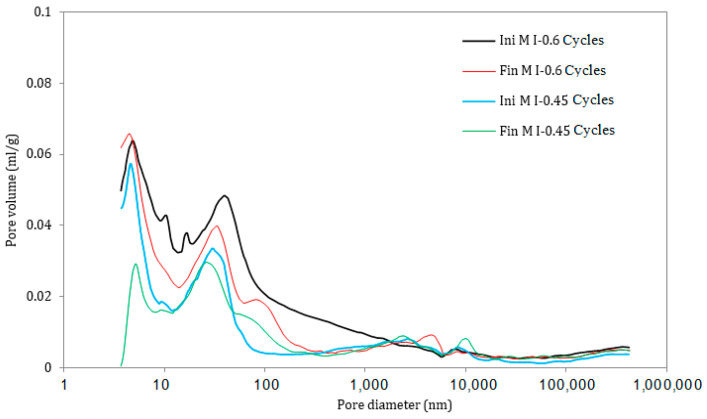
Pore size distribution in the samples under drying/wetting cycles, before (Ini) and after (Fin) ESA.

**Figure 16:**
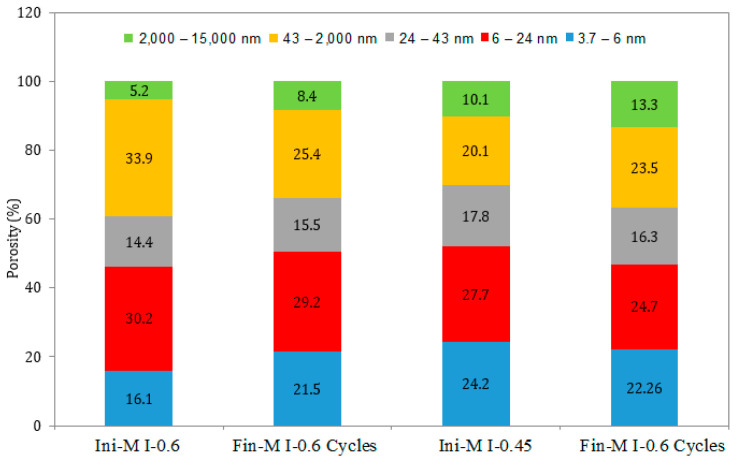
Variation in the pore volume of the samples during drying/wetting cycles, before (Ini) and after (Fin) ESA.

A comparison of the pore size distribution in the surface layer of the mortar samples (the first 1 mm of the sample) exposed to all the exposure conditions for 12 months in the attacking solution is illustrated in Figure 11, Figure 12, Figure 13, Figure 14, Figure 15 and Figure 16. The initial state represents the distribution of the pore size after 28 days of curing in water, whereas the final state represents the distribution of the pore size after 12 months of the ESA. The M I-0.6 samples experienced a significant decrease in the pore volume, between 43 nm and 2000 nm in terms of the pore diameter, when exposed to full immersion (Figure 11 and Figure 12). On the other hand, the variations in pore volume for the M I-0.45 prisms showed a slight decrease observed between 3.7 nm and 6 nm, caused by filling the gel pores with expansive products, without causing serious damage. It was confirmed by the porosity percentages distributed within the different ranges of pore sizes that the microstructural changes were more prominent in the samples mixed with high w/c ratios. For example, the porosity distribution in the M I-0.45 samples remained almost unchanged before and after the ESA. From 3.7 nm to 2000 nm, very slight decreases were observed. There was an increase in porosity from 10.06% to 14.6% between 2000 nm and 15,000 nm, which is associated with the formation of minor microcracks. The M I-0.6 samples showed relatively more significant changes, particularly between 43 nm and 2000 nm. There was a 28% decrease in porosity at this level, which is attributed to the precipitation of expansive products, particularly ettringite (Figure 11 and Figure 12). This comparison indicates that the w/c ratio has a significant influence on resistance to an ESA, which is consistent with previous findings (visual inspection, mass, expansion, compressive strength, and WAPTs) that have demonstrated the importance of using a lower w/c ratio to improve resistance to ESAs.

In semi-immersion conditions, the porosity percentage in the zone between 43 nm and 2000 nm decreased significantly, particularly in the M I-0.6 samples, going from 33.94% to 21.46% (Figure 13 and Figure 14). As previously stated, this decrease within the gel and capillary pores is most likely related to ettringite precipitation. In a similar manner to full immersion, the M I-0.45 samples experienced an increase in macropores between 2000 nm and 15,000 nm, as a result of the formation of microcracks. In both full immersion and semi-immersion conditions, increasing the w/c ratio from 0.45 to 0.6 resulted in more damage to the microstructure by the ESA.

Furthermore, the pore size distribution for the M I-0.45 and M I-0.6 samples subjected to continuous drying and wetting cycles show similar trends exhibited by the samples in full immersion and semi-immersion conditions. There was a significant decrease in the pore volume and porosity (from 33.94% to 25.44%) for the M I-0.6 samples between 43 nm and –2000 nm, as well as an increase in macro-porosity (between 2000 nm and 15,000 nm) for both w/c ratios (Figure 15 and Figure 16). This ranges from 5.29% (before the ESA) to 15.66% (semi-immersion) and 13.31% (drying/wetting cycles) after the ESA.

The MIP results for all the considered mixes were directly influenced by the w/c ratio rather than the exposure conditions. In the zone between 43 nm and 2000 nm, the compositions with a higher water content experienced a significant drop in pore volume, regardless of the type of accelerated ESA. The results confirm that the resistance of mortar to sulfate ingress decreases as the w/c ratio increases from 0.45 to 0.6. It should also be noted that the main variations in the pore structure during an ESA occur within gel pores and portlandite dissolution ranges. These findings are consistent with some studies [31,32,33] that indicate that the most expansive products (ettringite) originate in capillary and gel pores within the range of 1 nm to 1000 nm. Furthermore, these variations may be due to the dissolution of portlandite occurring in the 2000–3000 nm range, and the appearance of cracks in the 3000–15,000 nm range [34].

## 4. Discussion

The results in this study clearly show that even if the visual aspect of the specimens after an ESA is completely different, the kinetics of expansion remain practically the same for the mortars with either of the w/c ratios (0.45 and 0.6). The visual inspections conducted on the mortar bars showed that the highest degree of deterioration was reached in the samples cast with a high w/c ratio (w/c = 0.6). This behavior is attributed to the presence of a higher capillary volume, causing more salt crystallization and surface damage [35]. Despite the literature [36] indicating that only two stages occurred for all mortar mixes, a three-stage process was identified in this study for all the mortar mixes. Moreover, it seems interesting to correlate the expansion and mass variations, as the relationship followed a three-stage behavior process: stage 1 with a slight increase in the expansion and mass due to the penetration of sulfate ions and the beginning of the formation of expansive products, and stage 2 with a fast increase in expansion, associated with a high mass loss rate. In this stage, the samples enter the damaged range, when the material becomes more sensitive to the stresses (crystallization pressure) exerted by the formed crystals inside the pores. And stage 3, with significant expansion, inducing macroscopic damage and a loss of material. At the end of this stage, the mass loss stabilizes, indicating that the material has reached the failure range.

The mechanical strength results are consistent with the visual observations, which clearly demonstrate the high level of damage suffered by the mixes, particularly the M I-0.6 prisms. Since these samples exhibited high expansion and significant mass loss, the compressive strength results were somewhat expected. Ettringite precipitation inside the material results in the reduction of strength and stiffness, which is one of the major aspects of degradation [37].

**Figure 17:**
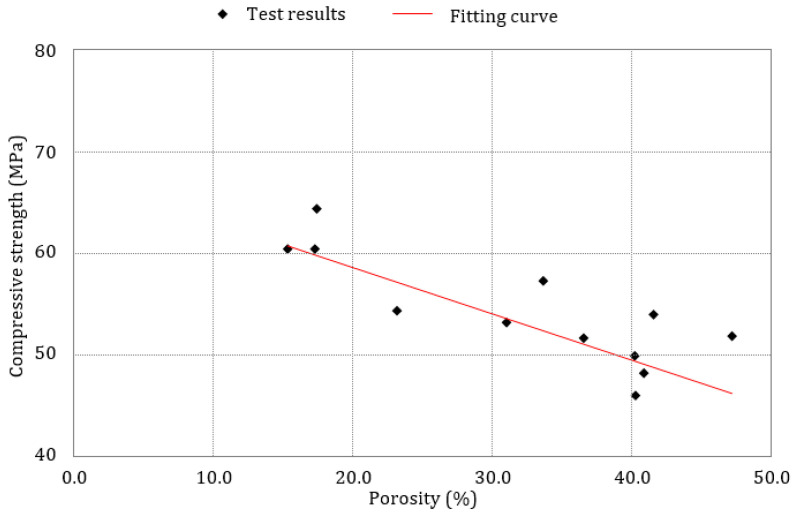
Relationship between porosity and compressive strength for M I-0.6 mortar samples exposed to ESA for all exposure conditions.

The porosity evolution during the ESA tests was consistent with the mechanical property results, length measurements, mass variation findings, and the visual observations. The M I-0.6 samples displayed low resistance to the ESA, due to a very high porosity percentage at the end of the accelerated attack, due to their chemical composition and high alumina content. Moreover, based on the expansion results, these samples reached the damage range after almost 7.5 months (30 weeks) of attack, which explains the presence of a high total porosity after week 30. Sulfate penetrates more quickly and more deeply through microcracks formed as a result of expansion stresses, thus the sample suffers from more expansion, surface scaling occurs, and the strength decreases, leading to the formation of more voids. Figure 17 illustrates the relationship between the compressive strength and porosity percentages of the M I-0.6 mortar samples exposed to the three different conditions. It can be seen that a correlation exists between porosity and compressive strength. A decrease in mechanical properties was accompanied by an increase in porosity during the ESA. In the early stages of the ESA, the compressive strength was at its highest value, while the porosity was at its lowest. At around 41% porosity, the compressive strength started to decrease and reached its lowest value (45 MPa). In the first three months following exposure to the ESA, the filling of the pores by ettringite, as well as salt crystals, increased the strength of the mortar samples and reduced the percentage of voids. With the progression of the ESA, expansive forces caused by the newly formed products and stresses caused by sulfate crystallization started to affect and damage the pore walls, increasing the porosity percentage and reducing the mechanical strength [28].

Finally, the MIP results are consistent with WAPT results. The major changes in the pore volume at the surface of the sample occur in terms of the pore diameter, with values between 43 nm and 2000 nm. Ettringite precipitates in gel and capillary pores located within this zone. In addition, portlandite dissolution, which promotes more ettringite formation, occurs between 2000 nm and 3000 nm, which explains the increase in porosity in this range during the ESA. Accordingly, ettringite precipitation is believed to be responsible for the decrease in porosity experienced by the M I-0.6 samples.

## 5. Conclusions

Based on the overall results obtained for the mortar samples during the considered exposure period, the following conclusions are drawn:

- ESA performance can be improved by mixing mortar with a medium w/c ratio (0.45). A reduced w/c ratio results in improved macro-structural and microstructural performance against an ESA. Reduced w/c ratio mixes demonstrated limited sulfate ingress and therefore less damage, as well as moderate expansion rates, slight mass loss variations, and better mechanical resistance against induced stresses. Microstructurally, the changes in pore volumes were limited compared to those observed with a higher w/c ratio;
- Sulfate penetration kinetics are not affected by the type of exposure to an ESA. For the same w/c ratio, the three exposure conditions studied (full immersion, semi-immersion, and drying/wetting cycles) produce very similar results when evaluating the expansion, mass, mechanical properties, WAPT porosity, and MIP. At this stage, it is difficult to distinguish between the three attacking conditions and determine which is faster or better. It should be noted, however, that each type of exposure results in a different type of degradation;
- Contrary to what is stated in the literature, we found that the expansion mechanism occurs as a result of a three-stage process. During stage 1, expansive products precipitate in large voids without causing significant expansion (expansion remains low and stable). During stage 2, ettringite begins to generate excessive stresses due to expansion. During the third and final stage, the expansion causes microcracks to develop, strength loss, and macropore formation, which ultimately lead to failure;
- Based on the WAPT method, total porosity is correlated with variations in compressive strength. Both exhibit a two-stage behavior. In the first stage of the process, the compressive strength increases, whereas the porosity decreases. Pores and capillaries of hardened pastes are filled by the products. At the second stage, there is a significant reduction in compressive strength, accompanied by an increase in total porosity. During the attack, the compressive strength of the surface decreases as a result of the formation of microcracks, which expand and cause scaling. In this case, the high porosity and low compressive strength observed at the end of the attack are consistent with the severe macroscopic damage observed and indicate that the ESA-induced expansion produced new pores within the material;
- The MIP results indicate that major changes in porosity and pore volume distribution are observed in gel and capillary pore ranges at the surface layer. According to these findings, ettringite is the main product to form in gel and capillary pores of cement-based materials exposed to an ESA. The dissolution of portlandite results in a greater amount of ettringite being present in the system.

This work has shown that the mode of exposure of the samples to an ESA does not impact the expansion kinetics. Thus, to develop an aging test for ESAs, it is better to simplify the exposure conditions and take, for example, the case of total immersion in a laboratory as the simplest method to assess the behavior of different materials with regards to this pathology. This approach will be applied in the near future to other low-carbon cements in order to confirm that it can also be applied to these types of cement.

## Figures and Tables

**Table 1:** Chemical composition of CEM I cement determined by ICP-AES and TGA.

Chemical Composition (%)	CEM I
CaO	62.79
SiO_2_	20.38
Al_2_O_3_	4.30
Fe_2_O_3_	3.80
TiO_2_	0.24
MgO	1.25
SO_3_	3.46
S	0
K_2_O	0.73
Na_2_O	0.35
Ignition Loss	2.04

**Table 2:** The main cement clinker content, according to Bogue’s analysis [25].

Cement	C_3_S	C_2_S	C_3_A	C_4_AF
CEM I	57.05	14.99	7.91	8.9

## Data Availability

Data are contained within the article.
